# Molecular evolution of influenza B virus during 2011–2017 in Chaoyang, Beijing, suggesting the free influenza vaccine policy

**DOI:** 10.1038/s41598-018-38105-1

**Published:** 2019-02-21

**Authors:** Na Lei, Hai-bin Wang, Yu-song Zhang, Jian-hong Zhao, Yi Zhong, Yuan-jie Wang, Li-yong Huang, Jian-xin Ma, Qiang Sun, Lei Yang, Yue-long Shu, Shu-ming Li, Ling-li Sun

**Affiliations:** 1 Chaoyang District Center for Disease Prevention and Control, Beijing, 100021 China; 2 National Institute for Viral Disease Control and Prevention, Chinese Center for Disease Prevention and Control, Beijing, 102206 China; 3 School of Public Health (Shenzhen), Sun Yat-sen University, Guangdong, 510275 China

## Abstract

Two influenza B virus lineages, B/Victoria and B/Yamagata, are co-circulating in human population. While the two lineages are serologically distinct and TIV only contain one lineage. It is important to investigate the epidemiological and evolutionary dynamics of two influenza B virus lineages in Beijing after the free influenza vaccine policy from 2007. Here, we collected the nasopharyngeal swabs of 12657 outpatients of influenza-like illness and subtyped by real-time RT-PCR during 2011–2017. The HA and NA genes of influenza B were fully sequenced. The prevalence is the highest in the 6–17 years old group among people infected with influenza B. Yamagata-lineage virus evolved to two inter-clade from 2011–2014 to 2014–2017. The amino acids substitutions of HA1 region were R279K in strains of 2011–2014 and L173Q, M252V in strains of 2014–2017. Substitutions L58P, I146V were observed in HA1 region of Victoria-lineage virus in 2011–2012 and I117V, N129D were showed in 2015–2017. Phylogenetic analysis of NA showed Yamagata-Victoria inter-lineage reassortant occurred in 2013–2014. Influenza B mainly infect the school-aged children in Beijing and the free influenza vaccine inoculation does not seem to block school-age children from infection with influenza B. The antigen characteristics of circulating influenza B were different to the recommended vaccine strains. We concluded that the Victoria-lineage vaccine strain should been changed and the free influenza vaccine should be revalued.

## Introduction

Influenza virus poses a serious threat to public health by causing the annual epidemics and occasional pandemics. Influenza viruses are divided into influenza A, B, C and D virus based on the antigenic specificity of the nucleoprotein and matrix protein and influenza A and B virus co-circulated globally in a typical seasonal pattern^1–3^. Unlike influenza A which has a broad host range and caused pandemics by antigenic shift, influenza B usually causes local epidemic with no natural animal host (other than seals) and a slower mutation rate^4–6^. However, influenza B still contributes about one third of the global influenza disease burden. Many recent reports are indicated that influenza B is associated with serious illness, such as acute myocardial infarction and so on^7^. More seriously, influenza B caused 0.058% deaths for all-cause death in Southern China, which was higher than influenza A^8^. Despite the significance of influenza B to human health, their epidemiological characteristics and antigenic dynamics are far less studied compared to influenza A.

Influenza B was first isolated in 1940, designated as influenza B/Lee/40 and then was classified into two distinct lineages by the genetically relationship of the HA gene, represented by the prototype viruses B/Victoria/2/87 (Victoria-lineage) and B/Yamagata/16/88 (Yamagata-lineage) since 1983^9–11^. The Yamagata- and Victoria-lineage have been co-circulating globally and often alternating in regional dominance. Two lineages undergo a slower antigenic variation with genetic evolution of the HA and NA genes including nucleotide mutations, and reassortment^12,13^.

The primary measure to prevent influenza virus infection is vaccination. Trivalent vaccine (TIV) has been produced containing influenza A/H1N1 and A/H3N2 antigens and a single influenza B antigen since 1977, despite some studies said low level of cross-protection provided by immunization with vaccine containing antigen from a single influenza B lineage^14,15^. Since 2007, the municipal government of Beijing provided free influenza vaccination for adult residents more than 60 years old and 6–17 years old elementary and high school students (Beijing influenza vaccination program for middle school in 2007, http://zfxxgk.beijing.gov.cn/110088/qt33/2015–10/20/content_625915.shtml)^16,17^. However, the overall estimate of influenza vaccine effectiveness (VE) was 46.9% for the 2013–2014 season and 5.0% for the 2014–2015 season^18^. After this policy of free vaccine was implemented in Beijing, there was no continuous monitoring of the restrictions on influenza B infection and the influence to the pathogenic characteristics of influenza B. So, the continuous surveillance for influenza B in Beijing reflected the relationship between influenza vaccination rate and immune protective effect which imperative and has economic significance. The aim of this retrospective surveillance during the 2011–2017 seasons in Beijing was to assess the epidemiological and phylogenetic characteristics of influenza B from outpatients with influenza-like illness (ILI), and analyze the mismatch ratio between the circulating and vaccine strains.

## Results

### Pathogen spectrum of influenza cases from October 2011 to September 2017

**Figure 1:**
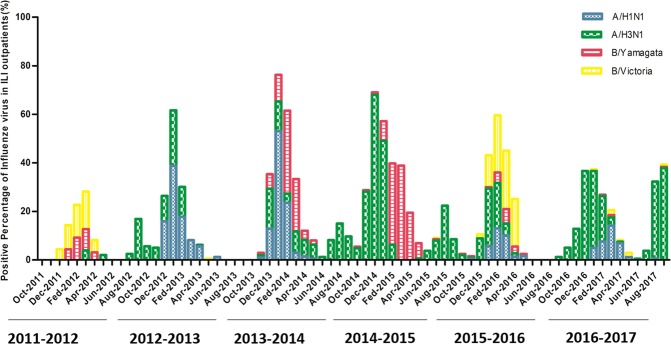
The percentage of influenza virus by real-time RT-PCR in surveillance seasons from 2011 to 2017. The y-axis represents positive percentage of influenza virus in assembled ILI outpatients every month. The x-axis shows month from October 2011 to September 2017. The period from October to September next year is defined as one influenza seasons. Blue boxes represent A/H1N1 virus, green boxes represent A/H3N2 virus, red boxes represent B/Yamagata-lineage, yellow boxes represent B/Victoria-lineage.

Total 12657 outpatients in the surveillance seasons from October 2011 to September 2017 were included in this study. Among them 446 (3.52%, from 0 to 7.8% per year) were positive for influenza A/H1N1; 1085 (8.57%, from 2.1% to 16.9% per year) were positive for influenza A/H3N2; 395 (3.12%, from 0 to 8.8% per year) were positive for influenza B/Yamagata-lineage; 246 (1.94%, from 0 to 7.2% per year) were positive for influenza B/Victoria-lineage. 3 (0.02%) were co-infected with two influenza viruses (Fig. 1). In total, 29.47% of influenza cases during the six years were infected by influenza B, which was higher than influenza A/H1N1, but lower than influenza A/H3N2. Notably, the positive percentage of influenza Yamagata-lineage in 2014–2015 was 7.2% that was higher than the other three influenza virus subtypes.

### Age distribution of influenza B

**Figure 2:**
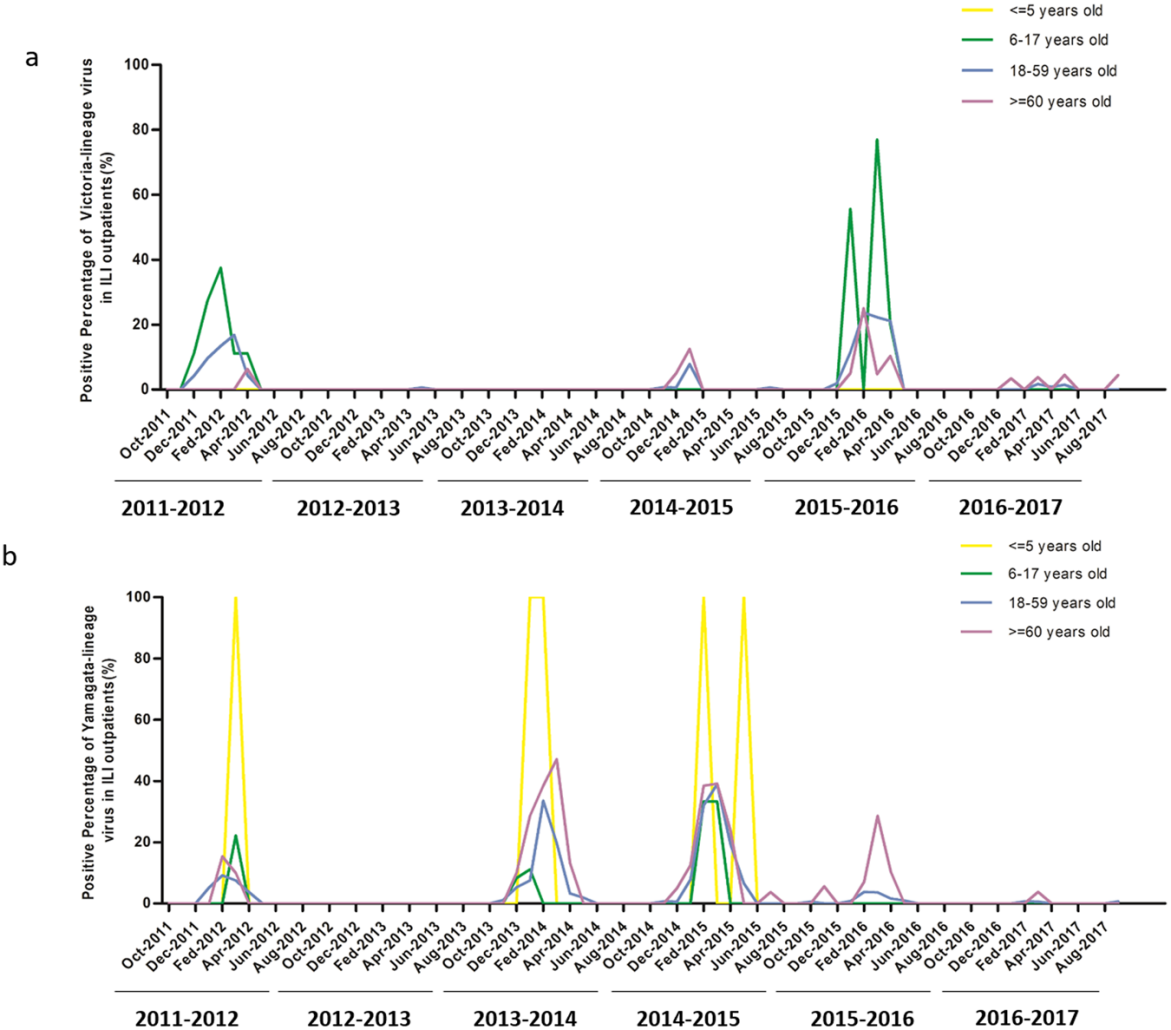
The age distribution of Yamagata- and Victoria-lineage virus. The y-axis represents the proportion of Victoria-lineage (**a**) and Yamagata-lineage (**b**) in assembled ILI outpatients caused by influenza virus every month. The x-axis shows month from October 2011 to September 2017. The period from October to September next year is defined as one influenza seasons. The age group ≤5 years old was shown by a yellow lines; the age group 6–17 years old was shown by a green lines; the age group 18–59 years old was shown by a blue lines and the age group ≥60 years old was shown by a violet lines.

The ILI cases were divided to four age groups (≤5 years old, 6–17 years old, 18–59 years old, ≥60 years old) in order to analyze the age distribution of influenza B. The positive rates of influenza B in four age groups were 17.24%, 4.99%, 4.82% and 6.71% (Table S1). Comparing with subtypes of influenza A, the prevalence of influenza B, especially for Yamagata-lineage, was higher in age group ≤5 year**s** old (χ^2^ test, *p* = 0.000). Although the prevalence of influenza B was lower than influenza A/H3N2, it was higher than influenza A/H1N1 in all age groups excepting ≤5 years old group. For all outpatients caused by influenza B, the positive percentage of Yamagata-lineage was the highest in ≤5 years old group. While the prevalence of Victoria-lineage virus was higher in age group 6–17 years old group than the other age groups (Fig. 2). Therefore, influenza B infection people concentrated in preschool children and school-aged children.

### Matching ratio of influenza B between seasonal viruses and vaccine strains

**Figure 3:**
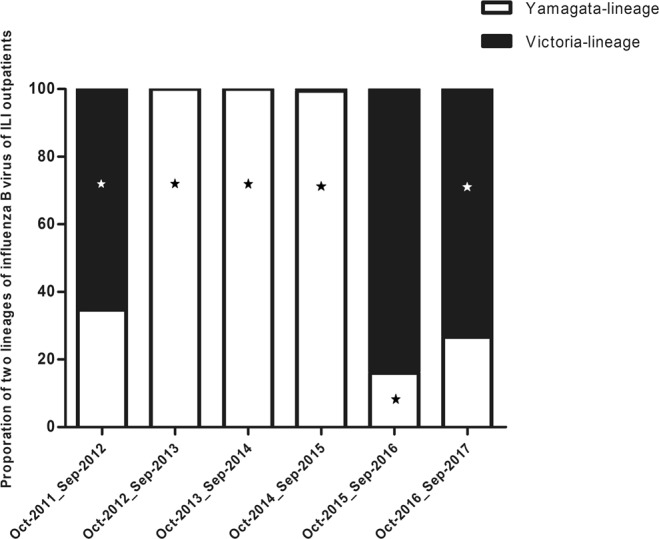
Distribution and recommended vaccine strain of influenza B in Beijing, 2011–2017. White boxes represent Yamagata-lineage; black boxes represent Victoria-lineage. Asterisks indicate the World Health Organization–recommended influenza B vaccine strain of that year. Data from October 2012 to September 2013 had only 1 case infected by influenza B virus.

During 2011–2017 surveillance seasons, Yamagata- and Victoria-lineage were infected synchronously or one lineage was in the ascendancy. But one lineage was recommended vaccine strain (Table S2). In 2011–2012 season, Victoria-lineage infection was predominated, but 34.68% of the circulating influenza B belonged to the opposite lineage to the northern hemisphere influenza B vaccine strains (B/Brisbane/60/2008). Subsequently, Yamagata-lineage was in dominant in 2012–2015 seasons and was consistent with recommended vaccine strain. Then Victoria-lineage was predominated in 2015–2016 season, however, the Yamagata-lineage (B/Phuket/3073/2013) was still recommended as the vaccine strain during this time span. Moreover, 26.67% of the circulating influenza B belonged to the opposite lineage to recommended vaccine strain in 2016–2017 season. On average, 31.20% of circling influenza B was mismatched to recommended influenza B vaccine strain during the consecutive six seasons (Fig. 3).

### Phylogenetic tree of the HA and NA of influenza B

The complete sequence of HA and NA gene were obtained from 83/110 (79.09%) of selected samples. Partial sequence of HA or NA sequence was excluded. A total of 83 full-length HA and 83 full-length NA sequences and 60 published sequences in GISAID and GenBank databases were analyzed for phylogenetic classification.

**Figure 4:**
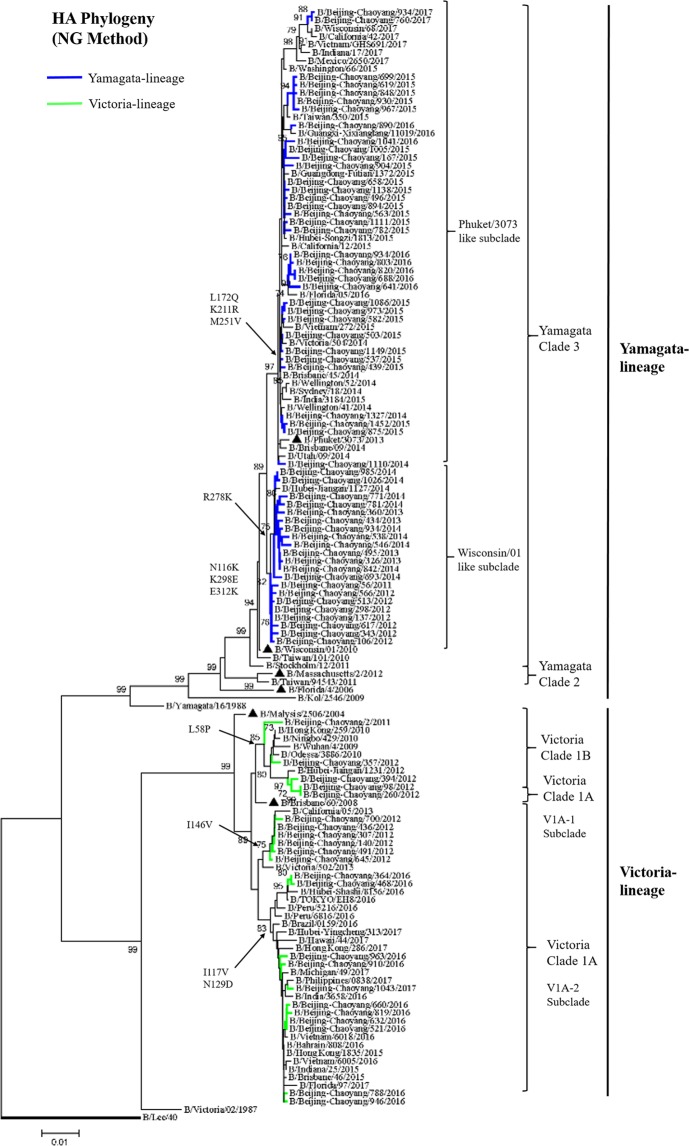
Phylogenetic tree of the HA gene sequences of influenza B viruses. HA sequences of influenza B viruses were compared with WHO recommended candidate vaccine (▲) and reference strains. The phylogeny was reconstructed using Neighbor-Joining (NG) method. Bootstrap values (>70%) and amino acid substitutions are mapped to key branches. Yamagata-lineage (blue), Victoria-lineage (green) are indicated.

Among 83 HA sequences, 22 (26.51%) of influenza B strains belonged to Victoria-lineage and 61 (73.49%) virus belonged to Yamagata-lineage based on the phylogenetic analysis of the HA sequence. Influenza B has been separated previously into three major antigenically distinct clades (clade 1, the B/Brisbane/60/2008 clade, clade 2, the B/Massachusetts/2/2012 clade, and clade 3, the B/Wisconsin/1/2010 clade), based on phylogenetic analysis of HA and NA genes^19^. Phylogenetic analysis showed that all HA sequences of Yamagata-lineage belonged to clade 3 (Fig. 4). Comparing to vaccine strain B/Wisconsin/1/2010, a series of amino acid changes were observed in the HA1 region of all strains: N116K, K299E and E313K. In detail, Yamagata-lineage evolved to two inter-clade from 2011–2014 seasons to 2014–2017 seasons. The Yamagata-lineage in 2011–2014 surveillance seasons shared special amino acids substitutions at R278K (9/33, 27.27%) (Tables 1, 2), whereas the Yamagata-lineage in 2014–2017 surveillance seasons shared amino acids substitutions at L172Q (34/34, 100%), K211R (8/34, 23.53%), M251V (26/34, 76.47%), which were related to B/Phuket/3073/2013 strain.

**Table 1:** The amino acid change sites of HA1 and NA of Victoria-lineage virus. Representative strain is randomly one of strains circulating in every surveillance season.

Representative strain	HA1							NA							
	58	117	129	146	171	184	189	41	42	45	120	220	295	340	358
*B/Brisbane/60/2008	**L**	**I**	**N**	**I**	**N**	**G**	**T**	**S**	**P**	**I**	**I**	**K**	**S**	**N**	**E**
B/Beijing-Chaoyang/2/2011	P	—	S	V	—	—	—	—	—	—	—	—	—	D	—
B/Beijing-Chaoyang/394/2012	P	—	S	V	D	E	—	P	S	—	—	N	—	D	A
B/Beijing-Chaoyang/167/2015	P	—	S	V	—	—	—	—	—	—	—	—	R	D	K
B/Beijing-Chaoyang/946/2016	—	V	D	—	—	—	A	—	—	T	V	N	R	D	K
B/Beijing-Chaoyang/1047/2017	—	V	D	—	—	—	—	—	—	—	—	N	R	D	A

* represents vaccine virus strains. “—” represents the same amino acids comparing to the same region of vaccine virus strains.

**Table 2:** The amino acid change sites of HA1 and NA of influenza B/Yamagata-lineage virus.

Representative strain	HA1							NA							
	116	172	211	251	278	298	312	41	42	45	49	62	73	295	342
*B/Wisconsin/1/2010	**N**	**L**	**K**	**M**	**R**	**K**	**E**	**S**	**Q**	**I**	**I**	**A**	**L**	**R**	**D**
B/Beijing-Chaoyang/56/2011	K	—	—	—	—	E	K	—	—	—	—	—	—	—	—
B/Beijing-Chaoyang/469/2012	K	—	—	—	—	E	K	—	—	—	—	—	—	—	—
*B/Massachusetts/2/2012	**N**	**L**	**K**	**M**	**R**	**K**	**E**	**S**	**Q**	**I**	**I**	**A**	**L**	**R**	**D**
B/Beijing-Chaoyang/326/2013	K	—	—	—	K	E	K	P	S	—	T	—	—	S	G
B/Beijing-Chaoyang/1327/2014	K	—	—	—	K	E	K	P	S	—	T	—	—	S	G
B/Beijing-Chaoyang/930/2015	K	Q	R	V	—	E	K	—	—	V	—	T	P	—	N
*B/Phuket/3073/2013	**K**	**L**	**K**	**M**	**R**	**E**	**K**	**S**	**R**	**V**	**I**	**A**	**P**	**S**	**D**
B/Beijing-Chaoyang/820/2016	—	Q	—	V	—	—	—	—	—	—	T	T	—	R	N
B/Beijing-Chaoyang/1240/2017	—	Q	—	V	—	—	—	—	—	—	M	T	—	R	N

* represents vaccine virus strains. “—” represents the same amino acids comparing to the same region of vaccine virus strains.

Phylogenetic analysis showed that all HA of Victoria-lineage were classified to clade 1. A portion of Victoria-lineage from 2011–2012 season was related to strain B/Brisbane/60/2008 and classified to clade 1B which had amino acids substitutions at L58P (8/8, 100%). All Victoria-lineage virus in 2011–2012 surveillance season shared amino acids substitutions at I146V (21/21), whereas Victoria-lineage virus in 2015–2017 surveillance seasons shared amino acids substitutions at I117V (23/29, 79.31%), N129D (29/29, 100%). They classified to clade 1 A within different inter-clades compared with vaccine strain B/Brisbane/60/2008.

**Figure 5:**
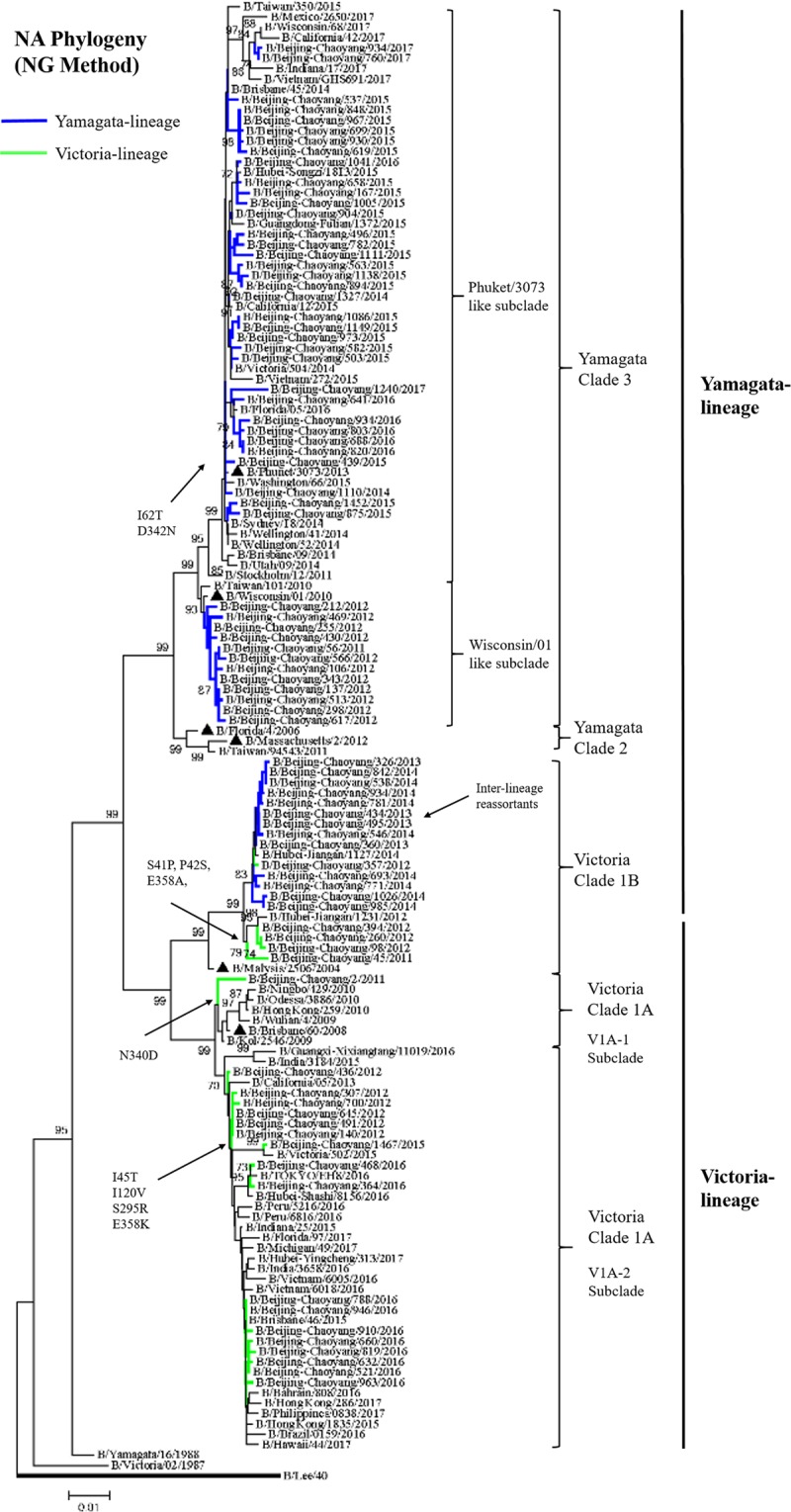
Phylogenetic tree of the NA gene sequences of influenza B viruses. NA sequences of influenza B viruses were compared with WHO recommended candidate vaccine (▲) and reference strains. The phylogeny was reconstructed using Neighbor-Joining (NG) method. Bootstrap values (>70%) and amino acid substitutions are mapped to key branches. Yamagata-lineage (blue), Victoria-lineage (green) are indicated.

Phylogenetic analysis of NA and HA showed Yamagata-Victoria inter-lineage reassortment occurred in 2013–2014 season (Fig. 5). HA of Yamagata-lineage in 2013–2014 season and NA of Victoria-lineage in 2011–2012 season formed new strains. The NA of Yamagata-lineage fall in clade 3. The Yamagata-lineage was the prevail lineage in 2014–2015 season and amino acids substitutions I45V, A62T, L73P of NA protein showed in most strains comparing to strain B/Wisconsin/1/2010 (Tables 1, 2). NA of Victoria-lineage classified into clade 1 and part of strains in 2011–2012 season developed an inter-clade different to 2015–2017 seasons. Comparing to strain B/Brisbane/60/2008, S41P, P42S substitutions in NA protein were showed in Victoria-lineage of 2011–2012 season, then strains of 2015–2017 seasons showed special amino acids substitutions I45T, I120V, D384G in NA protein.

### Antigenic analysis of HA1 region of Yamagata- and Victoria-lineage comparing to vaccine strain

Some receptor binding or in the antibody recognition in HA1 regions were observed in the 120 loop (position 116–137), the 150 loop (position 141–150), the 160 loop (162–167) and the helix 190 (194–202)^20,21^. Aligned HA1 of Yamagata-lineage to reference influenza B vaccine strain (B/Massachusetts/2/2012), only N116K substitutions were found in 2013–2015 seasons was on the 120 loop. Aligning HA of Victoria-lineage virus in HA1 region is different between 2011–2012 and 2015–2017 surveillance seasons when Victoria-lineage was predominant. The N129S and I146V substitutions were specially identified in 120 loop and 150 loop of HA1 region of Victoria-lineage in 2011–2012 surveillance season. Whereas, I117V and N129D substitutions were characteristically showed in 120 loop of HA1 region of Victoria-lineage in 2015–2017 surveillance seasons (Table 3).

**Table 3:** Amino acid substitutions found on the HA protein of influenza B viruses.

Influenza virus lineages	Epitope	Surveillance seasons^a^	Mutation^b^
B/Victoria-lineage	120-loop (116–137)	2011–2012	N129S (5/21)
		2015–2017	I117V (23/29), N129D (29/29)
	150-loop (141–150)	2011–2012	I146V (21/21)
B/Yamagata-lineage	120-loop (116–137)	2013–2015	N116K (43/44)

^a^Only amino acid substitutions shared by 50% of viruses or more in every season are reported in this table. Substitutions are compared with recommended vaccine strains in the same year. ^b^The frequency was the number of influenza viruses having the substitution to all the strains of influenza B virus in surveillance seasons.

## Discussion

In this study we described the epidemiology and phylodynamics of influenza B viruses during 2011–2017 surveillance seasons in Chaoyang District in Beijing. Epidemiological results of surveillance outpatients of ILI showed influenza B virus is more likely to infect people under 17 years old, who were preschool and school-aged children. The finding is consistent to the surveillance data of influenza B virus during 2009–2014 seasons in Shanghai and results from several community-based cohort studies that found that children were more frequently infected with B viruses than adults^22^. Influenza B contributed to a higher proportion (41.9%) among children and young teenagers and were associated with severe disease especially myocardial injury in children, which is observed in almost 70% of fatal cases^23–25^. Despite elementary and high school students can inoculate free influenza vaccines in Beijing, a recent report showed that the average coverage rate of TIV among students of the 43 schools in Beijing was 47.6% in 2014–2015 and children who received both 2013–2014 and 2014–2015 vaccinations had vaccine effectiveness of 29%^26^. VE against influenza B was higher among vaccinated individuals with no previous vaccination history was 75% compared with vaccinated individuals with a frequent vaccination history was 48% from 8 seasons^27^. Repeated previous vaccinations over multiple seasons had dose-dependent negative impacts on VE against both influenza A and B virus^28^. By this token, repeated vaccinations may low the VE of influenza vaccine. Contradictorily, the free influenza vaccine police may not the best protection to prevent infection of influenza B to school children. Further studies to confirm this finding are necessary.

Phylogenetic analysis of nucleotide and amino acids of influenza B virus is important in the assessment of antigenic evolvement. The evolution of the influenza B virus is driven by mutations and under positive selective pressure on the membrane-distal domain of HA1 in specific domains such as four epitopes, including loop-120, loop-150, loop-160 and helix 190^29^. Characterizing amino acid substitutions that occur along the trunk of Yamagata- and Victoria-lineage gene phylogenies, identifies changes that become fixed in the virus population across seasons. As expected, substitution N116K was key mutation in 2013–2015 seasons and caused Yamagata-lineage to be absolute prevalence. This mutation previously described to circulate in Italian in 2011–2015^30^. We also observed the 120-loop and 150-loop of HA1 of Victoria-lineage showed different mutations in 2011–2012 season and 2015–2017 seasons. The mutations of the antigenic site of HA1 make Victoria-lineage to be the absolute epidemic strain of these periods. Interestingly, we observed Yamagata/Victoria inter-lineage reassortment with HA of Yamagata-lineage and NA of Victoria-lineage, which was dominant in 2013–2014 seasons. The reassortment was reported in other researches and caused Yamagata-lineage to be dominated strain^31^. In conclusion, one or multiple mutation of four epitopes and subtypes reassortment may cause preponderant strain.

At the moment, TIV is still the effective measure to prevent infections and severe illnesses caused by influenza virus in the world^32,33^. However, the protective effect of vaccination is very weak^34^. The overall influenza VE for school children during the influenza season of 2016–2017 was 20.6% and estimated to 32% among risk groups and was 11% among the elderly^35^. The 3-seasons adjusted VE in preventing hospitalization as determined in a case-control study was 52% for influenza A and 28% for influenza B^36^. The reasons may consist three items for low protective rate. Firstly, TIV only include one lineage of influenza B and disparities from antigenic mismatches between the predominant circulating influenza B lineage in a given year and that year’s seasonal influenza trivalent vaccine. Our data showed 31.20% of infected by influenza B virus was mismatched to recommended influenza B vaccine strain. Recent surveillance data from the United States and Europe between 2001 and 2011 showed that 46–58% of mismatching between the vaccine and the predominant circulating lineage of influenza B virus^18^. Vaccine mismatch in 2015–2016 influenza season in Beijing was associated with an increased number of influenza school outbreaks^26^. Secondly, pathogenic diversity generated by shift and drift prevent vaccine strains from producing protective antibodies against epidemic strains^4,21^. The constant genetic and antigenic changes of influenza virus render them the ability to evade host immune system, thus limiting the vaccine effectiveness. Thirdly, cross-lineage serum antibodies were not detected on ferrets who were challenged with Victoria-lineage or Yamagata-lineage virus, though a significantly lower level of heterologous challenge virus in the respiratory tract was observed^37^. Hemagglutination-inhibition antibody titers indicate antibody responses against HA, which is not cross-reactive, and do not protect against mismatching influenza strains^38^. So, the need for development of quadrivalent influenza vaccines (QIV) containing Yamagata-lineage and Victoria-lineage antigens or engineered synthetic vaccine to encode two novel and broadly cross-protective monoclonal antibodies targeting influenza A and B are imperative^33,39,40^. The first QIV (a LAIV formulation) entered the market in 2012, and several other QIV formulations based on inactivated vaccine formulations, such as split and subunit formulations, have been licensed. The VE of QIV showed an immunogenicity and safety profile of the vaccine comparable with the two licensed trivalent vaccines containing the same strains^41^.

In conclusion, influenza virus that circulated in Chaoyang District in Beijing during the influenza seasons 2011–2017 evolved multiple antigenic epitope mutations, reassortment and belonged to different inter-clade comparing to recommended vaccine of influenza B. The epidemic strain of influenza B was the Victoria lineage mismatching to the vaccine strain Yamagata lineage. Though the free influenza vaccine for school-age children in Beijing, pre-school and primary school students still was in high infection by influenza B virus. The reasons may explained the phenomenon including antigenic variation, mismatching strains and repeated vaccination. So, the introduction of the QIV and broad-immunity vaccine are needed and the free vaccine policy in Beijing need to be revalued.

## Materials and Methods

### Ethics statement

The study was approved by the Ethics Review Committee of Chaoyang District Center for Disease Prevention and Control. All patients signed informed consent before the samples were collected. If the participants were children, their guardians were informed the consent.

### Sample collection

According to Influenza Surveillance Program for Beijing, the enrolment criteria for surveillance cases included: (a) outpatients were as ILI cases defined as fever with armpit temperature ≥38 °C accompanied by one of the general symptoms (i.e., cough, pharyngalgia or nasal congestion; (b) the clinical symptoms appeared at last 3 days; (c) patients have no antiviral treatment^42^. Forty nasopharyngeal or throat swabs were collected from the enrolled patients by 2 sentinel hospitals including China-Japan Friendship Hospital and Beijing Chao-Yang Hospital every week. Specimens were kept in 3 mL virus transport medium at 4–8 °C and sent to the network laboratory of Chaoyang District Center for Disease Prevention and Control within 24 h. The epidemiological information about the patients was input into the Chinese influenza Surveillance Net.

### Subtypes identification of influenza virus

Viral nucleic acids of all samples were extracted using QIAamp Viral RNA Mini Kit (Qiagen, Germany) according to the manufacturer’s instructions within 24 h after receiving the specimens and typed by real-time RT-PCR according to *Technical Guidelines for National Influenza Surveillance by Chinese National Influenza Centre* (http://www.chinaivdc.cn/cnic/fascc/201802/t20180202_158592.htm). Specimens were tested for influenza A and B viruses, then the positive samples were subtyped into A (H1N1) pdm09, H3N2, Victoria-lineage and Yamagata-lineage. Nasopharyngeal swabs which were positive for influenza viruses were cultured in Madin–Darby canine kidney cells for 4 to 5 days. The hemagglutination assay (HA) of cell culture was performed using 1% guinea pig erythrocyte. The culture medium with HA titer ≥1:8 was identified by hemagglutination inhibition method for subtypes of influenza virus.

### Sequencing and phylogenetic analyses of HA and NA of influenza B

Three (range 0–4) viral isolates identified as influenza B per month during 2011–2017 were randomly selected for sequencing. Viral nucleic acids were extracted from cell culture and the complete fragment of hemagglutinin (HA) gene and neuraminidase (NA) gene were sequenced. Specific primers used for target HA and NA gene amplification by RT-PCR were designed (Table S4), referencing to the HA and NA gene of strain B/Yamagata/16/88. The RT-PCR was performed using the One Step RT-PCR Kit (Qiagen, Germany) according to the handbook. The PCR products were analyzed using QIAxcel capillary electrophoresis (Qiagen, Germany) and sequenced by ABI 3730 DNA sequencer (PE-Applied Biosystems, Foster City, CA).

Multiple sequence alignment was performed using Clustal W (version 1.83). Genome sequences included in this study were submitted in the Global Initiative on Sharing Avian Influenza Data (GISAID)/GISAID accession numbers: EPI1153147-EPI1153378 (Table S4). A total of 83 HA and 83 NA sequences derived ILI outpatients from 2011 to 2017 were analyzed and 60 strains were downloaded from NCBI and GISAID using as reference strains in this study. The phylogenetic trees were constructed using MEGA software (version 6.06) applying the neighbor-joining method with 1000 bootstrap replicates.

### Statistical analysis

Data was analyzed using SPSS (Version 18.0; SPSS Inc., Chicago, IL, USA) and graphs drawing were used with GraphPad Prism software (Version 5.01; GraphPad, La Jolla, CA, USA). Difference between groups was evaluated using Pearson χ^2^ test or Fisher exact test.

## Supplementary information

Supplementary information

## References

[1] A revision of the system of nomenclature for influenza viruses: a WHO memorandum. *Bull World Health Organ* **58**, 585–591 (1980). PMC2395936 6969132

[2] Dowdle WR, Galphin JC, Coleman MT, Schild GC (1974). A simple double immunodiffusion test for typing influenza viruses. Bull World Health Organ.

[3] Ferguson L (2018). Influenza D Virus Infection in Feral Swine Populations, United States. Emerg Infect Dis.

[4] Bedford T (2015). Global circulation patterns of seasonal influenza viruses vary with antigenic drift. Nature.

[5] Chen JM (2007). Exploration of the emergence of the Victoria lineage of influenza B virus. Arch Virol.

[6] Bodewes R (2013). Recurring influenza B virus infections in seals. Emerg Infect Dis.

[7] Kwong JC (2018). Acute Myocardial Infarction after Laboratory-Confirmed Influenza Infection. N Engl J Med.

[8] Wang H (2014). Influenza associated mortality in Southern China, 2010–2012. Vaccine.

[9] Francis T (1940). A New Type of Virus from Epidemic Influenza. Science.

[10] Rota PA (1990). Cocirculation of two distinct evolutionary lineages of influenza type B virus since 1983. Virology.

[11] Hay AJ, Gregory V, Douglas AR, Lin YP (2001). The evolution of human influenza viruses. Philos Trans R Soc Lond B Biol Sci.

[12] Langat P (2017). Genome-wide evolutionary dynamics of influenza B viruses on a global scale. PLoS Pathog.

[13] Nakagawa N, Kubota R, Maeda A, Okuno Y (2004). Influenza B virus victoria group with a new glycosylation site was epidemic in Japan in the 2002–2003 season. J Clin Microbiol.

[14] Ambrose CS, Levin MJ (2012). The rationale for quadrivalent influenza vaccines. Hum Vaccin Immunother.

[15] Weir JP, Gruber MF (2016). An overview of the regulation of influenza vaccines in the United States. Influenza Other Respir Viruses.

[16] Yang J (2016). Seasonal influenza vaccination in China: Landscape of diverse regional reimbursement policy, and budget impact analysis. Vaccine.

[17] Lv M (2016). The free vaccination policy of influenza in Beijing, China: The vaccine coverage and its associated factors. Vaccine.

[18] Qin Y (2016). Influenza vaccine effectiveness in preventing hospitalization among Beijing residents in China, 2013–15. Vaccine.

[19] Chen R, Holmes EC (2008). The evolutionary dynamics of human influenza B virus. J Mol Evol.

[20] Wang Q, Cheng F, Lu M, Tian X, Ma J (2008). Crystal structure of unliganded influenza B virus hemagglutinin. J Virol.

[21] Ni F, Kondrashkina E, Wang Q (2013). Structural basis for the divergent evolution of influenza B virus hemagglutinin. Virology.

[22] Zhao B (2015). Epidemiological study of influenza B in Shanghai during the 2009-2014 seasons: implications for influenza vaccination strategy. Clin Microbiol Infect.

[23] Chan PK (2013). Influenza B lineage circulation and hospitalization rates in a subtropical city, Hong Kong, 2000–2010. Clin Infect Dis.

[24] Paddock CD (2012). Myocardial injury and bacterial pneumonia contribute to the pathogenesis of fatal influenza B virus infection. J Infect Dis.

[25] Gentile A (2018). Influenza virus: 16 years’ experience of clinical epidemiologic patterns and associated infection factors in hospitalized children in Argentina. PLoS One.

[26] Zhang L (2017). Influenza Vaccine Effectiveness in Preventing Influenza Illness Among Children During School-based Outbreaks in the 2014–2015 Season in Beijing, China. Pediatr Infect Dis J.

[27] Cheng AC (2017). Repeated Vaccination Does Not Appear to Impact Upon Influenza Vaccine Effectiveness Against Hospitalization With Confirmed Influenza. Clin Infect Dis.

[28] Saito, N. *et al*. Dose-Dependent Negative Effects of Prior Multiple Vaccinations against Influenza A and Influenza B among School Children: A Study of Kamigoto Island in Japan during the 2011/12, 2012/13 and 2013/14 Influenza Seasons. *Clin Infect Dis* (2018). 10.1093/cid/ciy202 29528389

[29] Shen J, Kirk BD, Ma J, Wang Q (2009). Diversifying selective pressure on influenza B virus hemagglutinin. J Med Virol.

[30] Tramuto F (2016). The Molecular Epidemiology and Evolutionary Dynamics of Influenza B Virus in Two Italian Regions during 2010-2015: The Experience of Sicily and Liguria. Int J Mol Sci.

[31] Oong XY (2017). Whole-Genome Phylogenetic Analysis of Influenza B/Phuket/3073/2013-Like Viruses and Unique Reassortants Detected in Malaysia between 2012 and 2014. PLoS One.

[32] Jamotte A, Chong CF, Manton A, Macabeo B, Toumi M (2016). Impact of quadrivalent influenza vaccine on public health and influenza-related costs in Australia. BMC Public Health.

[33] Soema PC, Kompier R, Amorij JP, Kersten GF (2015). Current and next generation influenza vaccines: Formulation and production strategies. Eur J Pharm Biopharm.

[34] Demicheli V, Jefferson T, Ferroni E, Rivetti A, Di Pietrantonj C (2018). Vaccines for preventing influenza in healthy adults. Cochrane Database Syst Rev.

[35] Souty C (2015). Early estimates of 2014/15 seasonal influenza vaccine effectiveness in preventing influenza-like illness in general practice using the screening method in France. Hum Vaccin Immunother.

[36] Sugaya N (2018). Three-season effectiveness of inactivated influenza vaccine in preventing influenza illness and hospitalization in children in Japan, 2013-2016. Vaccine.

[37] Kiseleva I (2018). Cross-Protective Efficacy of Monovalent Live Influenza B Vaccines against Genetically Different Lineages of B/Victoria and B/Yamagata in Ferrets. Biomed Res Int.

[38] Laurie, K. L. *et al*. Evidence for Viral Interference and Cross-reactive Protective Immunity Between Influenza B Virus Lineages. *J Infect Dis* (2018). 10.1093/infdis/jix509 PMC5853430 29325138

[39] Belshe RB (2010). The need for quadrivalent vaccine against seasonal influenza. Vaccine.

[40] Elliott STC (2017). DMAb inoculation of synthetic cross reactive antibodies protects against lethal influenza A and B infections. NPJ Vaccines.

[41] Graaf H, Faust SN (2015). Fluarix quadrivalent vaccine for influenza. Expert Rev Vaccines.

[42] Fang Q (2015). Molecular epidemiology and evolution of influenza A and B viruses during winter 2013–2014 in Beijing, China. Arch Virol.

