# Supplementary material for: Molecular evolution of influenza B virus during 2011–2017 in Chaoyang, Beijing, suggesting the free influenza vaccine policy
Source: Sci Rep. 2019 Feb 21;9:2432. doi: 10.1038/s41598-018-38105-1 (PMC6384887; doi:10.1038/s41598-018-38105-1)
Supplement: Supplementary file 1 — Supplementary information [file 41598_2018_38105_MOESM1_ESM.pdf]

Molecular evolution of influenza B virus during 2011-2017 in Chaoyang, Beijing, suggesting the free influenza vaccine policy

Na Lei[1, 2], Hai-bin Wang[1], Yu-song Zhang[1], Jian-hong Zhao[1], Yi Zhong[1], Yuan-jie Wang[1], Li-yong Huang[1], Jian-xin Ma[1], Qiang Sun[2, 3], Lei Yang[2], Da-yan Wang[2], Yue-long Shu[2, 3], Shu-ming Li[1*] and Ling-li Sun[1*]

**Age group**	**Total no. of enrolled ILI cases**	**Influenza B/Yamagata**	**Influenza B/Victoria**	**Influenza A/H1N1**	**Influenza A/H3N2**
≤5 years old	129	23 (17.83%)	1(0.77%)	2 (1.55%)	5 (3.88%)
6-17 years old	621	6 (1.00%)	25 (4.03%)	14 (2.25%)	48 (7.73%)
18-59 years old	10535	306 (2.90%)	200 (1.90%)	388 (3.68%)	855 (8.12%)
≥60 years old	1372	74 (5.39%)	18 (1.31%)	43 (3.13%)	171 (12.46%)

Table S1. Frequency of influenza virus subtypes in different age groups.

**Surveillance season**	**Influenza B lineage**	**Recommended strain**
2011-2012	Victoria	B/Brisbane/60/2008
2012-2013	Yamagata	B/Wisconsin/1/2010
2013-2014	Yamagata	B/Massachusetts/2/2012
2014-2015	Yamagata	B/Massachusetts/2/2012
2015-2016	Yamagata	B/Phuket/3073/2013
2016-2017	Victoria	B/Brisbane/60/2008

Table S2. Northern Hemisphere Influenza vaccine recommended influenza B strains of TIV.

Gene	Primers	Sequence (5´to 3´)[a]	Location[b]	Size (bp)	No. reference sequence in Genebank
HA	HA-F-18	ATGAAGGCAATAATTGTACTACT	18-1005	988	CY018765
	HA-R-1005	CTTTTGCATGTTCTCCTGTGTAGT			
	HA-F-832	GAACAATTGTCTATCAAAGAGGTGT	832-1836	1005	
	HA-R-1836	CGTTTCTTTGTAATGGTAACAAGCA			
NA	NA-F-1	AAACTGAGGCAAATAGGCCAAAAAT	1-867	867	CY018767
	NA-R-867	GCATGTGCATTCRTCAGTYTGTTRT			
	NA-F-651	AGAAGCATATACTGACACATACCAT	651-1501	851	
	NA-R-1501	AGTTCAGTAAGGACAATTGTTC			

Table S3. Primers used for amplification of HA and NA gene by RT-PCR. [a]Key to degenerated nucleotides: R = A + G, Y = C + T. [b]Based on nucleotide position of sequence from B/Yamagata/16/88.

EPI1153148	EPI1153147	B/Beijing-Chaoyang/2/2011	Victoria	2011-11-30	Sangon Biotech
EPI1153150	EPI1153149	B/Beijing-Chaoyang/45/2011	Victoria	2011-12-20	Sangon Biotech
EPI1153152	EPI1153151	B/Beijing-Chaoyang/56/2011	Yamagata	2011-12-28	Sangon Biotech
EPI1153154	EPI1153153	B/Beijing-Chaoyang/98/2012	Victoria	2012-01-10	Sangon Biotech
EPI1153156	EPI1153155	B/Beijing-Chaoyang/106/2012	Yamagata	2012-01-10	Sangon Biotech
EPI1153158	EPI1153157	B/Beijing-Chaoyang/137/2012	Yamagata	2012-01-16	Sangon Biotech
EPI1153160	EPI1153159	B/Beijing-Chaoyang/140/2012	Victoria	2012-01-17	Sangon Biotech
EPI1153162	EPI1153161	B/Beijing-Chaoyang/212/2012	Yamagata	2012-02-06	Sangon Biotech
EPI1153164	EPI1153163	B/Beijing-Chaoyang/255/2012	Yamagata	2012-02-10	Sangon Biotech
EPI1153166	EPI1153165	B/Beijing-Chaoyang/260/2012	Victoria	2012-02-10	Sangon Biotech
EPI1153170	EPI1153169	B/Beijing-Chaoyang/307/2012	Victoria	2012-02-14	Sangon Biotech
EPI1153172	EPI1153171	B/Beijing-Chaoyang/343/2012	Yamagata	2012-02-21	Sangon Biotech
EPI1153174	EPI1153173	B/Beijing-Chaoyang/357/2012	Victoria	2012-02-21	Sangon Biotech
EPI1153176	EPI1153175	B/Beijing-Chaoyang/394/2012	Victoria	2012-02-28	Sangon Biotech
EPI1153178	EPI1153177	B/Beijing-Chaoyang/430/2012	Yamagata	2012-03-06	Sangon Biotech
EPI1153180	EPI1153179	B/Beijing-Chaoyang/436/2012	Victoria	2012-03-06	Sangon Biotech
EPI1153182	EPI1153181	B/Beijing-Chaoyang/469/2012	Yamagata	2012-03-13	Sangon Biotech
EPI1153184	EPI1153183	B/Beijing-Chaoyang/491/2012	Victoria	2012-03-21	Sangon Biotech
EPI1153186	EPI1153185	B/Beijing-Chaoyang/513/2012	Yamagata	2012-03-21	Sangon Biotech
EPI1153188	EPI1153187	B/Beijing-Chaoyang/566/2012	Yamagata	2012-03-27	Sangon Biotech
EPI1153190	EPI1153189	B/Beijing-Chaoyang/617/2012	Yamagata	2012-04-03	Sangon Biotech
EPI1153192	EPI1153191	B/Beijing-Chaoyang/645/2012	Victoria	2012-04-10	Sangon Biotech
EPI1153194	EPI1153193	B/Beijing-Chaoyang/700/2012	Victoria	2012-04-17	Sangon Biotech
EPI1153377	/	B/Beijing-Chaoyang/326/2013	Yamagata	2013-11-25	Sangon Biotech
EPI1153196	EPI1153195	B/Beijing-Chaoyang/360/2013	Yamagata	2013-11-27	Sangon Biotech
EPI1153198	EPI1153197	B/Beijing-Chaoyang/434/2013	Yamagata	2013-12-10	Sangon Biotech
EPI1153200	EPI1153199	B/Beijing-Chaoyang/495/2013	Yamagata	2013-12-24	Sangon Biotech
EPI1153202	EPI1153201	B/Beijing-Chaoyang/538/2014	Yamagata	2014-01-06	Sangon Biotech
EPI1153204	EPI1153203	B/Beijing-Chaoyang/546/2014	Yamagata	2014-01-06	Sangon Biotech

EPI1153206	EPI1153205	B/Beijing-Chaoyang/671/2014	Yamagata	2014-01-21	Sangon Biotech
EPI1153208	EPI1153207	B/Beijing-Chaoyang/693/2014	Yamagata	2014-01-27	Sangon Biotech
EPI1153210	EPI1153209	B/Beijing-Chaoyang/771/2014	Yamagata	2014-02-09	Sangon Biotech
EPI1153212	EPI1153211	B/Beijing-Chaoyang/781/2014	Yamagata	2014-02-09	Sangon Biotech
EPI1153214	EPI1153213	B/Beijing-Chaoyang/842/2014	Yamagata	2014-02-17	Sangon Biotech
EPI1153216	EPI1153215	B/Beijing-Chaoyang/934/2014	Yamagata	2014-03-11	Sangon Biotech
EPI1153218	EPI1153217	B/Beijing-Chaoyang/985/2014	Yamagata	2014-03-17	Sangon Biotech
EPI1153220	EPI1153219	B/Beijing-Chaoyang/1026/2014	Yamagata	2014-03-25	Sangon Biotech
EPI1153224	EPI1153223	B/Beijing-Chaoyang/1233/2014	Victoria	2014-04-28	Sangon Biotech
EPI1153226	EPI1153225	B/Beijing-Chaoyang/1327/2014	Yamagata	2014-05-08	Sangon Biotech
EPI1153228	EPI1153227	B/Beijing-Chaoyang/439/2015	Yamagata	2015-01-08	Sangon Biotech
EPI1153232	EPI1153231	B/Beijing-Chaoyang/503/2015	Yamagata	2015-01-20	Sangon Biotech
EPI1153234	EPI1153233	B/Beijing-Chaoyang/537/2015	Yamagata	2015-01-27	Sangon Biotech
EPI1153236	EPI1153235	B/Beijing-Chaoyang/563/2015	Yamagata	2015-02-02	Sangon Biotech
EPI1153238	EPI1153237	B/Beijing-Chaoyang/582/2015	Yamagata	2015-02-02	Sangon Biotech
EPI1153240	EPI1153239	B/Beijing-Chaoyang/619/2015	Yamagata	2015-02-10	Sangon Biotech
EPI1153242	EPI1153241	B/Beijing-Chaoyang/658/2015	Yamagata	2015-02-16	Sangon Biotech
EPI1153244	EPI1153243	B/Beijing-Chaoyang/699/2015	Yamagata	2015-02-24	Sangon Biotech
EPI1153246	EPI1153245	B/Beijing-Chaoyang/782/2015	Yamagata	2015-03-10	Sangon Biotech
EPI1153248	EPI1153247	B/Beijing-Chaoyang/848/2015	Yamagata	2015-03-23	Sangon Biotech
EPI1153250	EPI1153249	B/Beijing-Chaoyang/875/2015	Yamagata	2015-03-23	Sangon Biotech
EPI1153252	EPI1153251	B/Beijing-Chaoyang/894/2015	Yamagata	2015-03-30	Sangon Biotech
EPI1153254	EPI1153253	B/Beijing-Chaoyang/904/2015	Yamagata	2015-03-30	Sangon Biotech
EPI1153256	EPI1153255	B/Beijing-Chaoyang/930/2015	Yamagata	2015-04-06	Sangon Biotech
EPI1153258	EPI1153257	B/Beijing-Chaoyang/967/2015	Yamagata	2015-04-13	Sangon Biotech
EPI1153260	EPI1153259	B/Beijing-Chaoyang/973/2015	Yamagata	2015-04-13	Sangon Biotech

EPI1153262	EPI1153261	B/Beijing-Chaoyang/1005/2015	Yamagata	2015-04-13	Sangon Biotech
EPI1153264	EPI1153263	B/Beijing-Chaoyang/1086/2015	Yamagata	2015-05-04	Sangon Biotech
EPI1153266	EPI1153265	B/Beijing-Chaoyang/1111/2015	Yamagata	2015-05-04	Sangon Biotech
EPI1153268	EPI1153267	B/Beijing-Chaoyang/1138/2015	Yamagata	2015-05-12	Sangon Biotech
EPI1153270	EPI1153269	B/Beijing-Chaoyang/1149/2015	Yamagata	2015-05-12	Sangon Biotech
EPI1153272	EPI1153271	B/Beijing-Chaoyang/1452/2015	Yamagata	2015-05-12	Sangon Biotech
EPI1153274	EPI1153273	B/Beijing-Chaoyang/1467/2015	Yamagata	2015-07-06	Sangon Biotech
EPI1153276	EPI1153275	B/Beijing-Chaoyang/167/2015	Yamagata	2015-11-23	Sangon Biotech
EPI1153278	EPI1153277	B/Beijing-Chaoyang/364/2016	Victoria	2016-01-06	Sangon Biotech
EPI1153280	EPI1153279	B/Beijing-Chaoyang/468/2016	Victoria	2016-01-13	Sangon Biotech
EPI1153282	EPI1153281	B/Beijing-Chaoyang/521/2016	Victoria	2016-01-27	Sangon Biotech
EPI1153284	EPI1153283	B/Beijing-Chaoyang/632/2016	Victoria	2016-02-10	Sangon Biotech
EPI1153286	EPI1153285	B/Beijing-Chaoyang/641/2016	Yamagata	2016-02-10	Sangon Biotech
EPI1153288	EPI1153287	B/Beijing-Chaoyang/660/2016	Victoria	2016-02-17	Sangon Biotech
EPI1153290	EPI1153289	B/Beijing-Chaoyang/688/2016	Yamagata	2016-02-24	Sangon Biotech
EPI1153292	EPI1153291	B/Beijing-Chaoyang/788/2016	Victoria	2016-03-09	Sangon Biotech
EPI1153294	EPI1153293	B/Beijing-Chaoyang/803/2016	Yamagata	2016-03-16	Sangon Biotech
EPI1153296	EPI1153295	B/Beijing-Chaoyang/819/2016	Victoria	2016-03-16	Sangon Biotech
EPI1153298	EPI1153297	B/Beijing-Chaoyang/820/2016	Yamagata	2016-03-16	Sangon Biotech
EPI1153300	EPI1153299	B/Beijing-Chaoyang/890/2016	Yamagata	2016-03-30	Sangon Biotech
EPI1153302	EPI1153301	B/Beijing-Chaoyang/910/2016	Victoria	2016-03-30	Sangon Biotech
EPI1153304	EPI1153303	B/Beijing-Chaoyang/934/2016	Yamagata	2016-04-06	Sangon Biotech

EPI1153306	EPI1153305	B/Beijing-Chaoyang/946/2016	Victoria	2016-04-06	Sangon Biotech
EPI1153308	EPI1153307	B/Beijing-Chaoyang/963/2016	Victoria	2016-04-13	Sangon Biotech
EPI1153310	EPI1153309	B/Beijing-Chaoyang/1041/2016	Yamagata	2016-04-20	Sangon Biotech
EPI1153312	EPI1153311	B/Beijing-Chaoyang/760/2017	Yamagata	2017-02-08	Sangon Biotech
EPI1153314	EPI1153313	B/Beijing-Chaoyang/934/2017	Yamagata	2017-03-15	Sangon Biotech
EPI1153316	EPI1153315	B/Beijing-Chaoyang/1043/2017	Victoria	2017-04-05	Sangon Biotech
/	EPI1153378	B/Beijing-Chaoyang/1240/2017	Yamagata	2017-05-03	Sangon Biotech

Table S4. Information about the influenza B strains selected for sequencing. "/" Represents no sequence.
